# Preparation of Sweet Potato Porous Starch by Marine Dextranase and Its Adsorption Characteristics

**DOI:** 10.3390/foods13040549

**Published:** 2024-02-10

**Authors:** Yue Hao, Mingwang Liu, Hao Ni, Yue Bai, Qingfang Hao, Lei Zhang, Xinxin Kang, Mingsheng Lyu, Shujun Wang

**Affiliations:** 1 Jiangsu Key Laboratory of Marine Bioresources and Environment/Jiangsu Key Laboratory of Marine Biotechnology, Jiangsu Ocean University, Lianyungang 222005, China; yuehao@jou.edu.cn (Y.H.); mwliu@jou.edu.cn (M.L.); haoniyoung@163.com (H.N.); yuebai@jou.edu.cn (Y.B.); qfhao@jou.edu.cn (Q.H.); leizhang@jou.edu.cn (L.Z.); kangxinxin@jou.edu.cn (X.K.); mslyu@jou.edu.cn (M.L.); 2 Co-Innovation Center of Jiangsu Marine Bio-Industry Technology, Jiangsu Ocean University, Lianyungang 222005, China

**Keywords:** dextranase, sweet potato porous starch, curcumin, proanthocyanidins

## Abstract

Dextranase (EC 3.2.1.11) is primarily applied in food, sugar, and pharmaceutical industries. This study focuses on using a cold shock *Escherichia coli* expression system to express marine dextranase SP5-Badex; enzyme activity increased about 2.2-fold compared to previous expression. This enzyme was employed to produce sweet potato porous starch, with special emphasis on the pore size of the starch. The water and oil adsorption rates of the porous starch increased by 1.43 and 1.51 times, respectively. Extensive Fourier transform infrared spectroscopy and X-ray diffraction revealed that the crystal structure of the sweet potato starch was unaltered by enzymatic hydrolysis. The adsorption capacities of the porous starch for curcumin and proanthocyanidins were 9.59 and 12.29 mg/g, respectively. Notably, the stability of proanthocyanidins was significantly enhanced through their encapsulation in porous starch. After 2.5 h of ultraviolet irradiation, the free radical scavenging rate of the encapsulated proanthocyanidins remained at 95.10%. Additionally, after 30 days of sunlight exposure, the free radical scavenging rate of the encapsulated proanthocyanidins (84.42%) was significantly higher than that (24.34%) observed in the control group. These research findings provide substantial experimental evidence for preparing sweet potato porous starch using marine dextranase.

## 1. Introduction

Dextranase (EC 3.2.1.11) is an enzyme commonly applied in food [1], sugar making [2], medicine [3], and biotechnology fields [4]. It exhibits specific hydrolytic activity toward α-1,6 glycosidic bonds [5]. Based on their structural characteristics, dextranases are classified into GH49 and GH66 families. Notably, dextranase derived from marine bacteria has high enzymatic efficiency at moderate temperatures, which makes it advantageous for producing highly polymerized iso-malt-oligosaccharides and the removal of dental plaque biofilms. However, there are few reports on marine-derived dextranases. The expression levels of this enzyme are low in wild bacteria, which necessitates heterologous expression as a vital means for enhancing its expression and facilitating scale-up preparation [6]. Dextranase is stably expressed in various host bacteria, and the GH66 family includes Cadex2870 dextranase obtained from the marine bacterium *Catenovulum* sp. DP03 [3].

Porous starch (PS) serves as a safe, economical, and natural adsorbent material in the biotechnology filed. The PS preparation methods principally involve physical [7], chemical [8], and biological enzymatic methods [9]. Of them, the enzymatic method is considered environmentally friendly. Previously, α-amylase starch was mostly used for preparing porous maize amylopectin, which effectively enhanced the water and oil adsorption rates of starch [10]. Subsequently, the combination of α-amylase and saccharase composite enzymes further increased the adsorption rate of porous maize starch [11]. However, the same enzyme exhibits varied effectiveness when used with different types of starch. For instance, the oil adsorption rate of the α-amylase and saccharase composite enzyme used with rhubarb rice starch increased to 174% [12]. Therefore, selecting effective enzymes is crucial for processing different types of porous starch [13]. Currently, PS preparation from sweet potato using dextranase has not been studied.

PS can provide protection and slow release of the adsorbed substances. Proanthocyanidins (OPCs) and curcumin (CUR) are polyphenol polymers common in fruits and vegetables. OPCs are easily absorbed by humans, and have substantial dimer scavenging capacity for free radicals [14], and various health benefits such as preventing urinary tract infections [15], treating obesity [16], and improving hypertension [17]. CUR exhibits anticancer [18], anti-inflammatory [19], antitumor [20], and antioxidant [21] properties. However, OPCs contain multiple phenolic hydroxyl groups, which are sensitive to light, pH, and temperature; therefore, they are prone to oxidation and decomposition [22]. CUR, on the other hand, has negative properties such as poor water solubility, low adsorption, fast metabolism, and low bioavailability [23]. Using PS for embedding both OPCs and CUR can partially address these issues.

In this study, the marine dextranase SP5-Badex was heterologously expressed in *Escherichia coli* (BL21) with cold shock promoter. Then, the application of dextranase to prepare porous starches was investigated. The sweet potato porous starch was characterized and used for encapsulating OPCs and CUR. The porous materials could be used as a protective agent to improve the stability of environmentally sensitive drugs. The study provides a foundation for applying dextranase and sweet potato porous.

## 2. Materials and Methods

### 2.1. Materials

The dextranase-producing strain *Bacillus aquimaris* SP5 was stored in our laboratory (Jiangsu Key Laboratory of Marine Biotechnology, Lianyugang, China) [24]. *Xho*I and *Sac*I enzymes were purchased from NEW ENGLAND Biolabs Inc. (Beijing, China). The pColdI vector and *E. coli* BL21 (DE3) receptor cells were purchased from Takara Bio Inc. (Shanghai, China). Sweet potato starch, tapioca starch, wheat starch, kudzu starch, barely porous starch and yam starch were purchased from Xuzhou Renheju Food Factory (Xuzhou, China), Xinliang Grain and Oil Co. (Fujin, China), Zhangjiajie Xiang’amei Food Co. (Zhangjiajie, China), Luzhong Food Co. (Jinan, China), and Wuzhi County Xinnongyuan E-commerce Co. (Jiaozuo, China), respectively. OPCs were purchased from Shanghai Macklin Biochemical Technology Co. (Beijing, China). CUR and 2,2-diphenyl-1-picrylhydrazyl radical were purchased from Aladdin Holdings Group Co. (Beijing, China).

### 2.2. Methods

#### 2.2.1. Cloning and Expression of the Marine Dextranase SP5-Badex

The dextranase SP5-Badex target gene was extracted from *B. aquimaris* SP5, and primers were designed to introduce double cleavage sites at *Xho*I and *Sac*I sites for PCR amplification of the enzyme gene [24]. The target gene was ligated into the pColdI vector containing the cold shock promoter. The recombinant plasmid prepared was transformed into *E. coli* BL21 (DE3) receptor cells, and the enzyme was expressed. The enzyme activity was measured. Finally, SP5-Badex protein was purified using an AKTA protein purifier (GE AKTA GO, GE, Boston, MA, USA).

##### SP5-Badex Purification

*E. coli* BL21 (DE3) receptor cells containing SP5-Badex protein were fermented and cultured, and induced by the addition of Isopropyl-beta-D-thiogalactopyranoside (IPTG) at a final concentration of 10 mM, 15 °C for 24 h. The broth was centrifugated at 8000× *g*, 15 min. The precipitates were washed three times using phosphate buffer saline (PBS) buffer at 100 mM, pH 7.4, then ultrasonically crushed to obtain the crude enzyme solution. The target protein was purified by nickel column, and the protein concentration of the crude enzyme solution and the purified enzyme solution was determined by the bicinchoninic acid assay (BCA) method. Detection was carried out by SDS-PAGE electrophoresis.

##### SP5-Badex Activity Assay

One hundred fifty microliters of 3% dextran T70 solution was mixed with 50 μL of enzyme solution to react 15 min at 40 °C. Two hundred microliters 3,5-Dinitrosalicylic acid (DNS) was added. Then, it was boiled for 5 min, and 3 mL of pure water was added. The reaction solution was detected at 540 nm. The enzyme activity (U/mL) was calculated according to Formula (1). The dextranase activity (1 Unit) is defined as the amount of dextranase required to produce 1 μmol of glucose per minute by hydrolyzed dextran T70.

(1) $$dextranase\;enzyme\;activity\left(U/mL\right)=\frac{W_{1}\;\times \;D}{W_{2}\;\times \;t\;\times \;V}$$

In Equation (1), *W*_1_ is quality of reducing sugars (μg), *D* is dilution, *W*_2_ molecular weight of glucose (g/mol), *t* is reaction time (min), and *V* is enzyme liquid volume (mL).

##### Properties of SP5-Badex

The optimal temperature and stability of SP5-Badex at 20–60 °C were studied, and the optimum pH and stability of SP5-Badex at pH 4.0–9.0 were studied. For the temperature stability, the dextranase was incubated for 2 h, and the residual enzyme activity was measured every 20 min. The pH stability was determined by placing the dextranase in a buffer solution at pH 4.0–9.0 for 1h and then the residual enzyme activity was determined.

#### 2.2.2. Porous Starch Preparation

The preparation process is shown in Figure S1. Eleven common starches, including barley starch, broad bean starch, yam starch, potato starch, corn starch, acorn starch, sweet potato starch, wheat starch, batata starch, tapioca starch and kudzu starch, were selected, and 20 mL, 100 mM, pH 5.5 PBS buffer solution was added, respectively, and then put into a 50 °C water bath for a wet heat treatment for 20 min and then added with an equal volume of enzyme solution at 30 °C for a reaction of 48 h. Preliminary screening was carried out on different kinds of porous starch by the results of scanning electron microscopy and the water/oil adsorption rate of porous starch.

The starch obtained through primary screening was subjected to secondary screening using the modified PS preparation method of Rashmi Singh [25]. The different starches were sampled separately and dissolved in pH 5.5, and 100 mM disodium hydrogen phosphate-citric acid buffer solution (10% *w*/*v*). Ultrasonication was performed for 30 min, and enzyme solution equal to the volume of buffer solution was added to the starch solution for different times. After the reaction was terminated by adding 95% ethanol solution, PS was obtained through drying (75 °C), grinding, and sieving.

#### 2.2.3. Comparison of Different Types of Porous Starch

##### Scanning Electron Microscopy (SEM)

The samples were weighed and applied to the surface of a conductive tape and covered with gold powder. Then, SEM images were observed using a scanning electron microscope (JSM-6390LA, Nihon Electronics, Tokyo Japan) at 50 kV accelerating voltage and 10,000× magnification.

##### The Water/Oil Adsorption Rate of Porous Starch

An amount of 0.5 g of PS was weighed in a centrifuge tube and 5 mL of pure water/oil was added. The mixture was vortex mixed for 1 min, allowed to stand for 30 min, and centrifuged at 10,000× *g* for 8 min. The supernatant was discarded. The water/oil adsorption rate was calculated using the method of Jung, Y. [26]. The formula is given below:

(2) $$water/oil\;adsorption\;rate\;(\%)=\frac{W_{3}-W_{2}-W_{1}}{W_{1}}\times 100$$

In Equation (2), *W*_1_ denotes the mass of PS, *W*_2_ represents the weight of the centrifuge tube, and *W*_3_ is the total weight of PS and the centrifuge tube after centrifugation.

#### 2.2.4. Characteristics of the Sweet Potato Porous Starch

##### Fourier Transform Infrared Spectroscopy (FT–IR)

First, 300% (*w*/*w*) potassium bromide was added to the samples and ground thoroughly. The samples were then pressed into tablets. The transmittance at 4000–400 cm^−1^ was determined by placing the samples in a Fourier transform infrared spectrometer (Thermo Fisher Scientific, Waltham, MA, USA), and the atmospheric background was deducted after completing the scanning, and the change in the peaks was observed.

##### X-ray Diffraction (XRD)

The powdered samples were scanned using an X-ray diffractometer (X’Pert Powder, PANalytical AG, Almelo, The Netherlands) at 30 mA current and 40 kV voltage in the range of 10°–40°.

#### 2.2.5. Loading Capacity of PS with CUR/OPCs

CUR (10/20 mg) was dissolved in 100 mL anhydrous ethanol solution. And the OPCs (10/20 mg) were dissolved in 5 mL ethanol. Then, PS was added to the mixture, and the mixture was ultrasonicated for 10 min. The precipitate was collected after centrifugation (10,000× *g*, 5 min).

The loading amount was determined using Wang’s [27] method with slight modifications. The mixture of CUR/OPCs-PS was ultrasonic treated for 10 min then it was centrifuged (10,000× *g*, 5 min). The absorbance of the supernatant was measured at 450 nm (CUR) and 500 nm (OPCs) to determine the change in concentration, respectively. The loading amounts were calculated using the following equations.

(3) $$loading\;amount\;(mg/g)=\frac{{(C}_{0}-C_{1})\times V}{M}$$

In Equation (3), *C*_0_ is the initial concentration of OPCs-anhydrous ethanol solution, *C*_1_ is the concentration of OPCs after adsorption by porous starch, *V* is the volume of the supernatant, and *M* is the mass of porous starch.

#### 2.2.6. The Protective Effect of PS on OPCs

##### Ultraviolet Irradiation Stability of OPCs

OPC-PS and OPCs were placed under ultraviolet light for different periods. Then, both were dissolved in ethanol and then reacted with DPPH (1,1-diphenyl-2-picrylhydrazyl radical) avoiding light for 30 min. The change in absorbance at 517 nm was determined, and the free radical scavenging rate was determined according to the following equation [28]:

(4) $$DPPH\;free\;radical\;scavenging\;rate\;(\%)=(1-\frac{O_{2}-O_{3}}{O_{1}})\times 100$$

In Equation (4), *O*_1_ represents the absorbance of ethanol mixed with DPPH, *O*_2_ denotes the absorbance of the OPC-PS sample solution mixed with DPPH, and *O*_3_ is the absorbance of the OPC-PS sample solution mixed with ethanol.

##### Storage Stability of OPCs

OPCs and OPC-PS were placed outdoors under natural light for 30 days. The DPPH radical scavenging rate was measured to compare changes in their antioxidant capacity over time.

#### 2.2.7. Data Analysis

All the experiments in this study were established with three parallel experimental groups. The analysis of variance was performed using SPSS based on the mean and standard deviation of the three experimental groups during statistical analyses. *p* < 0.05 indicates a significant difference in the data between the two groups.

## 3. Results and Discussion

### 3.1. Cloning and Expression of Marine Dextranase SP5-Badex

**Figure 1:**
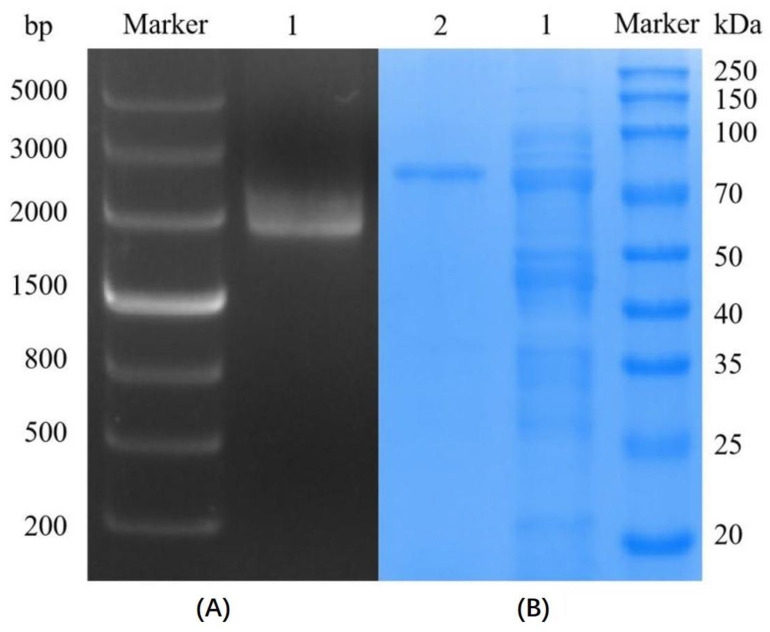
Cloning and expression of SP5-Badex (**A**) 1% agarose gel electrophoresis. Lane 1 has the target gene after double digestion, (**B**) 10% SDS-PAGE. Lane 1 has the crude enzyme, and lane 2 has the pure enzyme.

The position of bands in lane 1 in the graph for 1% agarose gel electrophoresis in Figure 1A revealed that the target gene (1722 bp) was successfully obtained through double cleavage using SacI and XhoI loci. The target gene was ligated into the pColdI vector of the cold shock *E. coli* expression system, transformed into *E. coli* BL21 (DE3) receptor cells, and expressed (Figure 1B). Lanes 1 and 2 contain the crude and pure enzyme solutions, respectively. The target protein size was 66 kDa, and the band position proved that SP5-Badex was successfully expressed.

#### Study on Enzymatic Properties of SP5-Badex

**Figure 2:**
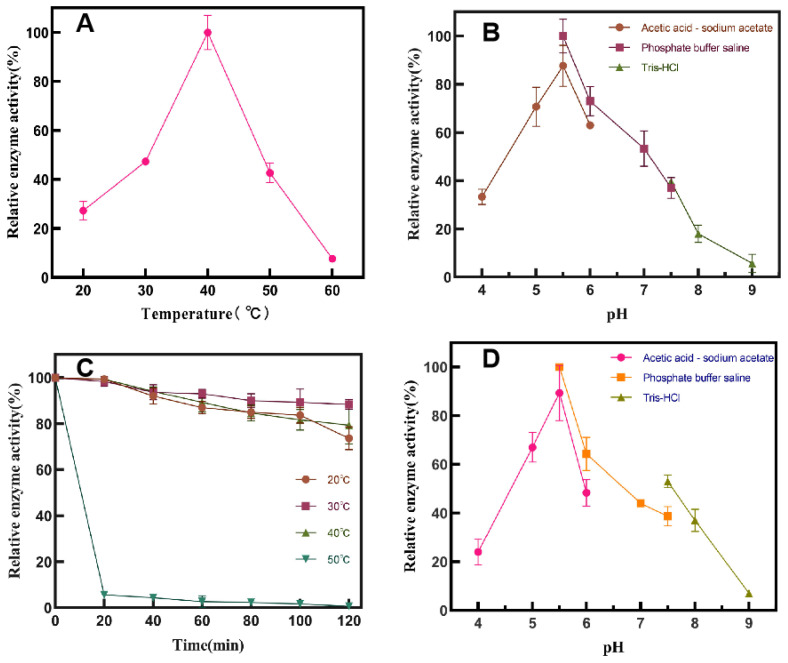
Activity of SP5-Badex was affected by (**A**) temperature, (**B**) pH, (**C**) temperature stability, (**D**) pH stability.

The optimum reaction temperature of SP5-Badex was 40 °C (Figure 2A), and the optimum pH was 5.5 (Figure 2B). The enzyme was stable at 40 °C (Figure 2C) and pH 5.5 (Figure 2D). The activity of SP5-Badex was increased 2.2-fold compared with a previous study [24]. The rapid cooling induction after attachment of the cold shock promoter cspA may favour the expression of the protein, which is also protected by the activation of molecular chaperone genes in the system [29].

### 3.2. Dextranase Hydrolyzed Different Starches

#### 3.2.1. SEM Images of Different PS

**Figure 3:**
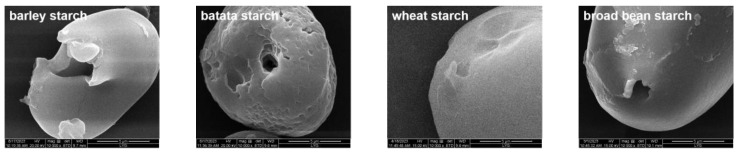

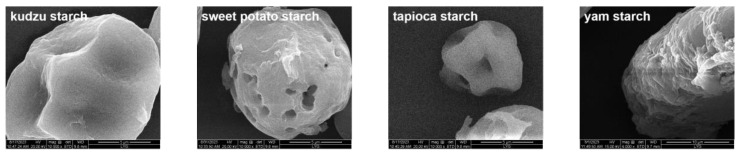
SEM images of different species of starches after enzymatic digestion.

The SEM image (Figure 3) revealed that the PS can be classified as macroporous, concave, and with small pores based on its morphology. SP5-Badex could perform enzymatic hydrolysis of many starch types, but the results were highly variable, with different pore sizes and distributions.

#### 3.2.2. The Water/Oil Adsorption Rate of Different PS

Due to the uneven distribution of starch pore size, the starches of different origin (barley, batata, broad bean, wheat, kudzu, sweet potato, tapioca, yam) were subjected to water and oil adsorption measurements. The results in Table 1 show a water adsorption of 103% and oil adsorption of 88% for sweet potato porous starch, and there are also more holes observed by SEM (Figure 3). The hydrophilicity and lipophilicity were found to be different for different types of starches. Therefore, it is possible to judge whether the prepared porous starch belongs to hydrophilic or amphiphilic materials and the suitable substances to be adsorbed based on the hydrophilicity and lipophilicity of the starch.

### 3.3. Effect of Treatment Time on Sweet Potato Starch

#### 3.3.1. SEM of Sweet Potato Starch with Different Enzyme Digestion Times

**Figure 4:**
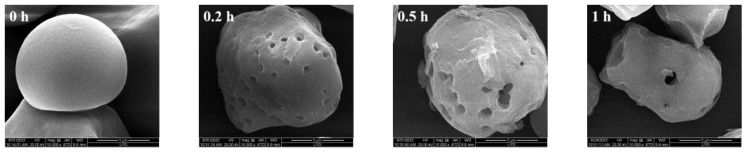

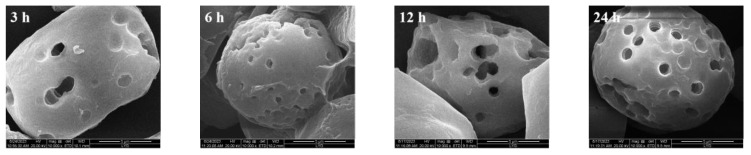
SEM images of SPS at different enzyme digestion times.

The effect of the enzyme on starch was more evident when the enzyme digestion time was prolonged (Figure 4). From the initial smooth surface of full starch granules, 10 min of enzymatic digestion alone produced fine pores. The pore size and pore capacity also increased significantly when the enzymatic digestion time was increased. This proves that the enzyme effectively acted on the starch’s internal structure and affected the morphology of its granules. The results were similar to those of Li Guo [13], who used glucoamylase, glycosylase, and α-amylase to obtain the synergistic effect of the three enzymes.

#### 3.3.2. The Water/Oil Adsorption Rate of PS

We determined the water and oil adsorption rates of PS (Table 2). The water and oil adsorption rates of PS increased as the time of starch enzymatic hydrolysis increased. Compared with starch without enzymatic digestion, the water adsorption rate (103%) increased 1.43 times and the oil adsorption rate (89%) increased 1.51 times at 24 h of enzymatic digestion. This result indicates that PS has better hydrophilicity, and enzymatic digestion led to a better and significant change in the oil adsorption of starch. No significant difference was observed between the water and oil adsorption rates at 12 and 24 h of enzymatic hydrolysis, which was consistent with the pore size results of the SEM images (Figure 4). The larger the pores and pore volume, the stronger the water and oil adsorption capacities. Wheat porous starch was also prepared using dextranase, and the water and oil adsorption rates of this starch were 80.12% and 78.23%, respectively [30]. The sweet potato porous starch had a stronger adsorption capacity.

### 3.4. Loading Amount of PS with CUR or OPCs

CUR adsorption in PS was determined. The loading increased as the amount of PS added increased, but the loading capacity was the highest at a CUR:PS ratio of 1:60. The amount of adsorption was 9.59 mg/g (Table 3). Due to the limited hole capacity, continued CUR addition did not increase the loading capacity. By contrast, the adsorption amount increased because the CUR mass that could be accommodated in the pores of the loaded PS was limited. This led to a conclusion similar to that of Wu L [31], which is that sweet potato porous starch can effectively load CUR.

OPCs were combined with PS according to different configuration ratios. Table 3 presents the results of load capacity and load amount of OPCs with PS. The maximum loading of OPCs (12.29 mg/g) was observed at a quality ratio of OPCs to starch of 1:60. Similar to the experimental findings of Xiaotong Bu [32], there exists a better loading release capacity of starch for OPCs. This indicates that PS exhibits a better adsorption capacity for OPCs. The PS showed a better adsorption capacity for OPCs than CURs. It might be related with the property of OPC/CUR. CUR is insoluble in water. CUR is more hydrophobic than OPC. OPCs are also antioxidants and light-sensitive. The freeze drying method has been used to immobilize OPCs on the starch granule surface in the food and health care field [33], but this method cannot protect OPC activity well. In this experiment, OPCs were encapsulated inside PS, which effectively resisted the external environment’s influence on OPCs and played a certain role in slow release.

### 3.5. Characterization of Porous Starch

**Figure 5:**
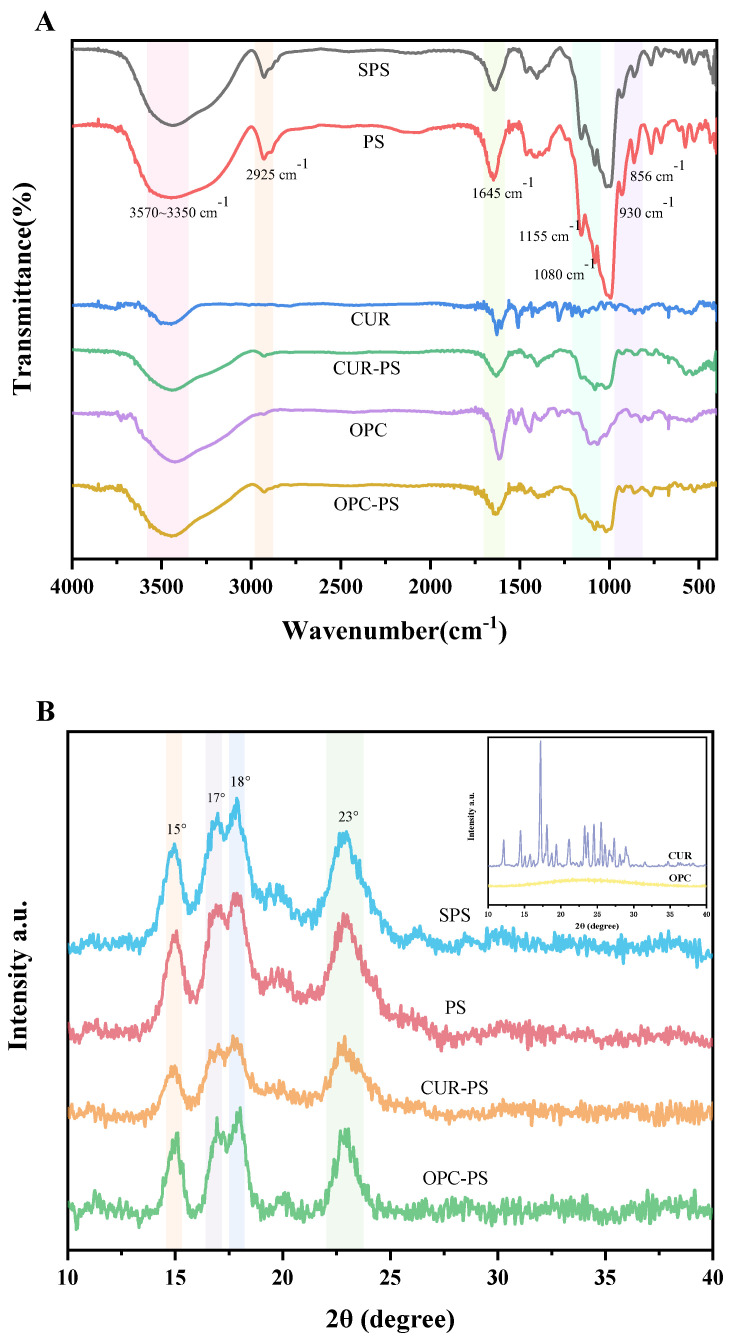
Characterization of sweet potato porous starch. (**A**) FT–IR spectra of SPS, PS, CUR, CUR–PS, OPCs, and OPC–PS, (**B**) X-ray diffractogram.

The Fourier transform infrared spectroscopy (FT–IR) showed that compared with PS, no new adsorption peaks appeared for SPS, and the positions of the characteristic peaks did not change (Figure 5A). This indicated that no chemical bonds were altered or added during PS preparation, either through ultrasonication or enzymatic treatment. The broader adsorption peaks of SPS and PS at 3350–3570 cm^−1^ were chiefly related to –OH stretching. The peak at 1645 cm^−1^ was generated by C=O stretching vibrations, and the two peaks were related to the hydrophilicity of the substances. The adsorption peak at 2925 cm^−1^, representing –CH_2_ bonding, was mainly related to lipophilicity, which indicated that enzymatic digestion effectively improved the water and oil adsorption rates of SPS (Table 1). Fingerprints at 1080 and 1155 cm^−1^ represented C–O and C–O–H groups, respectively, and the adsorption peaks at 1022 and 1047 cm^−1^ represented the amorphous and crystalline regions of starch, respectively [34]. IR 1047 cm^−1^/1022 cm^−1^ and IR 995 cm^−1^/1022 cm^−1^ represented the ordered structure and double helix structure of starch, respectively [35]. IR 1047 cm^−1^/1022 cm^−1^ of PS was slightly higher than that of SPS, suggesting that enzymatic degradation primarily acted on the amorphous region of starch, thereby increasing crystallinity. The experiments of L.D. Lacerda [36] also proved this point. The spectra of CUR exhibited distinct adsorption peaks at 3502, 1598, 1508, and 1280 cm^−1^ and were attributable to –OH, C=O, C–C, and aromatic C–O stretching vibrations, respectively [37]. The redshift of the characteristic peak from 1598 cm^−1^ to 1628 cm^−1^ and the disappearance of the characteristic peak near 3502 cm^−1^ into a broad peak following encapsulation of CUR by starch prove the occurrence of intermolecular interactions between CUR and starch. OPC–PS profiles revealed new adsorption peaks similar to those of OPC profiles at 3760–3925 cm^−1^, 1670–1890 cm^−1^, and 1440–1570 cm^–1^. This indicated that OPCs and PS were successfully bound following C–O–C interactions through different glucose units [38]. The experiments of Yan Zhang [39] aptly proved this aforementioned notion.

In summary, Han Xiuying [40] drew similar conclusions regarding the preparation of modified starch-loaded CUR, where OPCs and CUR, which are both phenolics, form hydrogen bonds with the hydroxyl groups of PS. Both OPCs and CUR interact with –OH and C–O of starch. When the drug characteristic peaks appeared in the PS profile and the groups of the PS itself were not destroyed, the drug was proved to be successfully encapsulated by PS.

The starch crystalline types are classified as more tightly structured type A [41], more loosely constructed type B [42], type C consisting of type A and type B [43], and amylose and lipid complex type V [44]. X-ray diffractogram (Figure 5B) revealed that the four starches have characteristic peaks at 15°, 17°, 18°, and 23°, which proved that SPS is a type A starch and that ultrasonication, enzymolysis, and drug loading does not change the crystal structure of starch. The crystallinity calculated based on the area of crystalline and amorphous regions of the XRD pattern was 26.28% for SPS and increased to 37.81% for PS because SP5–Badex hydrolyzed largely the amorphous regions of the starch and barely hydrolyzed the crystalline regions. This thus increased the relative crystallinity [45], and the same was also verified for the change in the moiety in the FT–IR spectrum (Figure 5A). The crystallinity of both CUR–PS and OPC–PS was lower than that of PS, and the drug characteristic peaks itself disappeared. This proved that both CUR and OPCs were loaded in the amorphous region of starch, so they would not change the PS crystal structure. CUR was encapsulated in the amorphous region of PS, which decreased the relative crystallinity [46]. In summary, both FT–IR and XRD results indicate that the drug was present in the amorphous region of the PS, which consequently made the drug more amenable to solubilization for later use.

### 3.6. The Protective Effect of PS on OPCs

**Figure 6:**
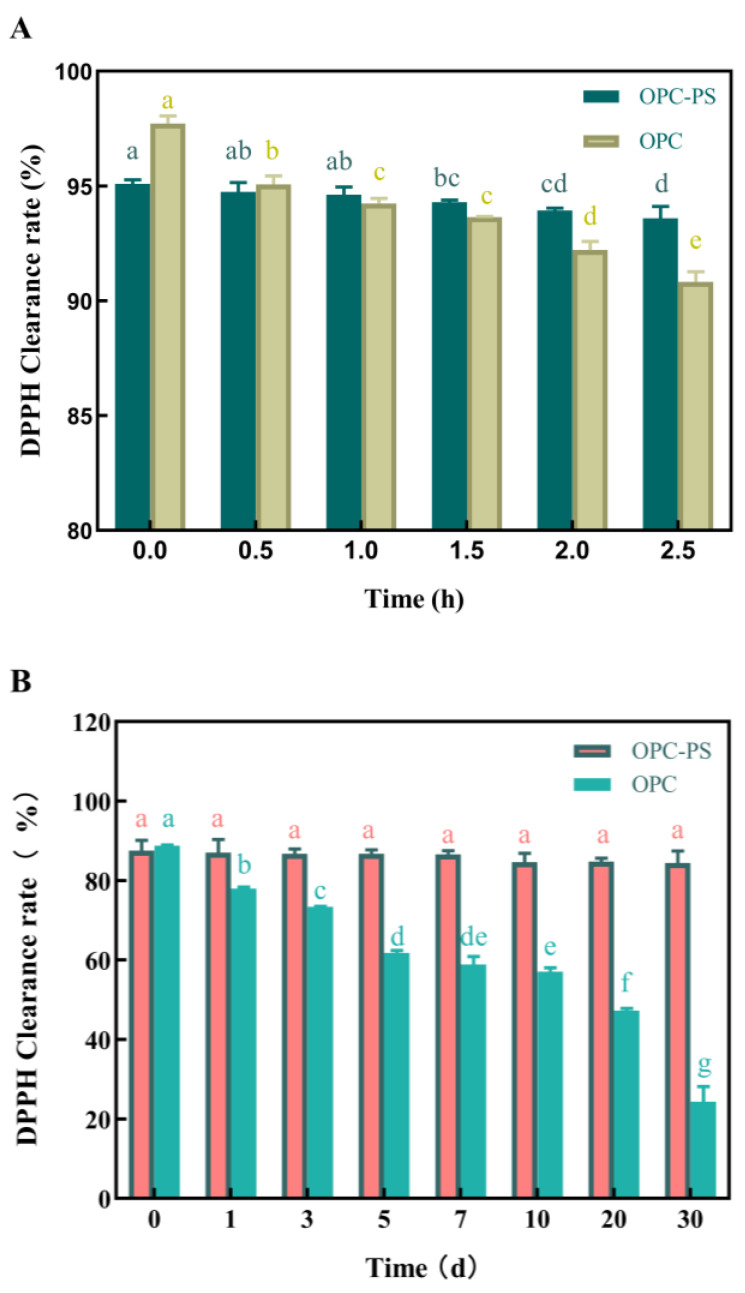
The effect of antioxidant activity of proanthocyanidins treated by (**A**) ultraviolet irradiation, (**B**) storage outdoors under natural light. Different letters within the same group indicate significant differences (*p* < 0.05).

As shown in Figure 6A, due to the slow release effect of PS. OPCs had a higher initial scavenging rate of DPPH free radicals (95.10%). However, with UV irradiation of OPCs for only 2.5 h, the free radical scavenging rate was reduced to 93.60%. The free radical scavenging ability of OPC–PS exhibited no obvious decreasing trend. Proanthocyanidins have strong absorption of UV [47]. However, from the analysis of the antioxidant results in Figure 6A, it is speculated that the antioxidant activity of proanthocyanidins decreases after absorption of UV, and OPC–PS inhibited the rate of decrease in antioxidant activity due to UV. Although OPCs have a high medicinal value, their use is limited by their light instability [48]. Under light-avoidance conditions, the initial DPPH radical scavenging rates of OPCs and OPC–PS were 88.78% and 86.76%, respectively (Figure 6B). The scavenging rate of OPC–PS was slightly lower than that of OPCs because of its slow release effect. The DPPH radical scavenging rate of OPCs at room temperature and under sunlight irradiation exhibited an evident decreasing trend, and it decreased to 24.34% by the thirtieth day. By contrast, OPC–PS exhibited almost no significant change at room temperature and under sunlight irradiation, and the DPPH radical scavenging rate remained at 84.42% on the 30th day. A study indicated [49] that the larger the pore volume of PS, the stronger the protection ability of OPCs. In addition, the number of pores and the hydrophilicity/oleophilicity of the starch are also important indicators for the selection of porous starch materials.

In summary, OPC–PS exhibited a more stable antioxidant activity than OPCs, even after UV and daily light irradiations, because PS not only acts as a slow release agent but also protects the embedded drug.

## 4. Conclusions

Marine-derived SP5-Badex can be expressed more efficiently and rapidly using the cold shock *E. coli* expression system. SP5-Badex specifically hydrolyzes the α-1,6 glycosidic bond, combined with the ultrasound-assisted method of hydrolyzing the amorphous region of sweet potato starch, and the porous starch of sweet potato is prepared. The sweet potato porous starch exhibited impressive water and oil adsorption rates of 103% and 89%, respectively. The structure of the amylose crystalline region in the sweet potato porous starch remained unaltered. Notably, OPCs and CUR were effectively adsorbed onto the sweet potato porous starch, with loading amounts of 12.29 and 9.59 mg/g, respectively. These drugs were absorbed in the amorphous region of the starch, which facilitated their easier solubilization. UV irradiation for 2.5 h resulted in a 6.88% reduction in free radical scavenging for unembedded OPCs, while only a 1.50% reduction was observed for the embedded OPCs. Furthermore, the free radical scavenging in the control group decreased by 64.43% after 30 days of sunlight exposure at room temperature, whereas OPC scavenging in the embedded group was only reduced by 3.09%. These findings demonstrate that after embedding, the sweet potato porous starch exhibited a protective effect on the drug. Consequently, these results build an experimental foundation for preparing sweet potato porous starch using marine dextranase.

## Figures and Tables

**Table 1:** Determination of water/oil adsorption rate of different PS.

Starch Type	Water Adsorption (%)	Oil Adsorption (%)
barley starch	51.79 ± 3.36 ^d^	67.86 ± 4.92 ^b^
batata starch	97.27 ± 4.33 ^ab^	69.85 ± 5.00 ^b^
broad bean starch	47.89 ± 1.05 ^d^	63.57 ± 5.21 ^b^
kudzu starch	92.84 ± 2.55 ^b^	70.54 ± 8.01 ^b^
sweet potato starch	102.70 ± 3.75 ^a^	88.29 ± 4.309 ^a^
tapioca starch	71.25 ± 3.42 ^c^	68.84 ± 4.29 ^b^
wheat starch	89.21 ± 4.87 ^b^	87.93 ± 3.72 ^a^
yam starch	69.01 ± 0.41 ^c^	87.24 ± 3.61 ^a^

All values are mean ± standard deviation. Different letters within the same column indicate significant differences (*p* < 0.05).

**Table 2:** Determination of water/oil adsorption rate of sweet potato porous starch.

Time of Enzymatic Digestion of Starch (h)	Water Adsorption (%)	Oil Adsorption (%)
0	72 ± 4.80 ^d^	59 ± 3.36 ^b^
0.2	85 ± 4.64 ^c^	74 ± 8.30 ^ab^
0.5	86 ± 2.78 ^c^	77 ± 11.83 ^ab^
1	86 ± 1.24 ^c^	78 ± 14.32 ^ab^
3	90 ± 5.99 ^bc^	80 ± 4.24 ^ab^
6	92 ± 3.70 ^bc^	82 ± 14.27 ^ab^
12	99 ± 6.12 ^ab^	84 ± 7.88 ^ab^
24	103 ± 3.75 ^a^	89 ± 4.30 ^a^

All values are mean ± standard deviation. Different letters within the same column indicate significant differences (*p* < 0.05).

**Table 3:** The loading amount of PS with CUR/OPC.

CUR/OPC:PS (*w*:*w*)	Loading Amount with CUR (mg/g)	Loading Amount with OPC (mg/g)
2:50	3.16 ± 0.35 ^c^	3.78 ± 0.34 ^c^
1:50	6.71 ± 0.40 ^b^	7.75 ± 0.87 ^b^
1:60	9.59 ± 0.57 ^a^	12.29 ± 2.68 ^a^
1:70	9.00 ± 0.32 ^a^	11.87 ± 0.39 ^a^
1:80	9.38 ± 0.28 ^a^	11.78 ± 0.43 ^a^

All values are mean ± standard deviation. Different letters within the same column indicate significant differences (*p* < 0.05).

## Data Availability

The original contributions presented in the study are included in the article and supplementary material, further inquiries can be directed to the corresponding author.

## Supplementary Materials

The following supporting information can be downloaded at: https://www.mdpi.com/article/10.3390/foods13040549/s1, Table S1: Abbreviation and full name list; Figure S1: Flow chart of preparation of the porous starches.
