# Supplementary material for: Preparation of Sweet Potato Porous Starch by Marine Dextranase and Its Adsorption Characteristics
Source: Foods. 2024 Feb 10;13(4):549. doi: 10.3390/foods13040549 (PMC10888179; doi:10.3390/foods13040549)
Supplement: Supplementary file 1 [file foods-13-00549-s001.zip › foods-2824280-supplementary.pdf]

Table S1. Abbreviation and full name list.

Reagent abbreviation	Full name
PS	porous starch
OPCs	proanthocyanidins
CUR	curcumin
IPTG	Isopropyl-beta-D-thiogalactopyranoside
PBS	phosphate buffer saline
BCA	bicinchoninic acid assay
DNS	3,5-Dinitrosalicylic acid
CUR-PS	curcumin-sweet potato porous starch
OPCs-PS	Proanthocyanidins-sweet potato porous starch

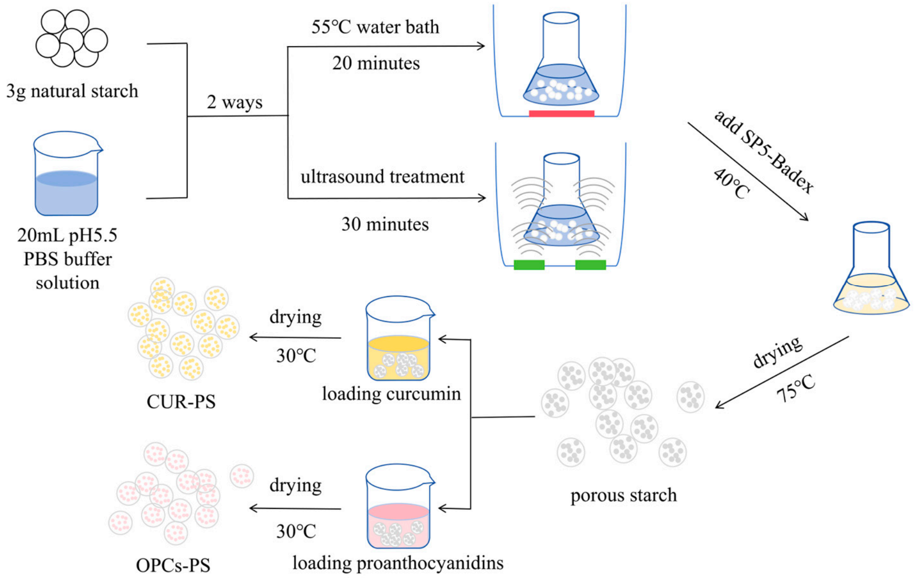

Figure S1. Flow chart of preparation of the porous starches.
